# Early Interneuron Dysfunction in ALS: Insights from a Mutant *sod1* Zebrafish Model

**DOI:** 10.1002/ana.23780

**Published:** 2012-12-31

**Authors:** Alexander McGown, Jonathan R McDearmid, Niki Panagiotaki, Huaxia Tong, Sufana Al Mashhadi, Natasha Redhead, Alison N Lyon, Christine E Beattie, Pamela J Shaw, Tennore M Ramesh

**Affiliations:** 1 Department of Neuroscience, Sheffield Institute for Translational Neuroscience, University of SheffieldSheffield, United Kingdom; 2 Department of Biology, University of LeicesterLeicester, United Kingdom; 3 Department of Neuroscience, Ohio State UniversityColumbus, OH; 4 Medical Research Council Centre for Developmental and Biomedical Genetics, University of SheffieldSheffield, United Kingdom

## Abstract

**Objective:**

To determine, when, how, and which neurons initiate the onset of pathophysiology in amyotrophic lateral sclerosis (ALS) using a transgenic mutant *sod1* zebrafish model and identify neuroprotective drugs.

**Methods:**

Proteinopathies such as ALS involve mutant proteins that misfold and activate the heat shock stress response (HSR). The HSR is indicative of neuronal stress, and we used a fluorescent *hsp70-DsRed* reporter in our transgenic zebrafish to track neuronal stress and to measure functional changes in neurons and muscle over the course of the disease.

**Results:**

We show that mutant *sod1* fish first exhibited the HSR in glycinergic interneurons at 24 hours postfertilization (hpf). By 96 hpf, we observed a significant reduction in spontaneous glycinergic currents induced in spinal motor neurons. The loss of inhibition was followed by increased stress in the motor neurons of symptomatic adults and concurrent morphological changes at the neuromuscular junction (NMJ) indicative of denervation. Riluzole, the only approved ALS drug and apomorphine, an NRF2 activator, reduced the observed early neuronal stress response.

**Interpretation:**

The earliest event in the pathophysiology of ALS in the mutant *sod1* zebrafish model involves neuronal stress in inhibitory interneurons, resulting from mutant Sod1 expression. This is followed by a reduction in inhibitory input to motor neurons. The loss of inhibitory input may contribute to the later development of neuronal stress in motor neurons and concurrent inability to maintain the NMJ. Riluzole, the approved drug for use in ALS, modulates neuronal stress in interneurons, indicating a novel mechanism of riluzole action. ANN NEUROL 2013;73:246–258

Neurodegenerative diseases including amyotrophic lateral sclerosis (ALS) are characterized by the presence of protein inclusions in the affected neurons. Emerging data indicate that protein misfolding may be of mechanistic importance in these diseases.^1^ Mutations in the ubiquitously expressed superoxide dismutase (*SOD1*) gene account for 20% of cases of the familial form of ALS. More than 150 mutations in the *SOD1* gene have been discovered, including the point mutations G93R and G85R.^2^ Recent studies also implicate *SOD1* in the sporadic form of ALS and suggest a prionlike propagation of misfolded SOD1.^3^–^5^ Interestingly, some of the newly identified genes implicated in ALS, such as *TARDBP* and *FUS*, are also proteins that show a high propensity to misfold and prionlike activity.^6^ However, we still do not know the precise mechanism by which mutant proteins cause toxicity.^5^,^7^ The emerging consensus view is that multiple interacting pathophysiological factors, including protein misfolding, contribute to the neuronal toxicity in ALS.^8^,^9^

Despite progress in revealing multiple molecular processes involved in disease pathology, relatively little is known about when and how the disekease, which starts focally, spreads throughout the motor network.^10^–^12^ Interestingly, even in the subtypes of ALS caused by SOD1 mutations, there is considerable phenotypic heterogeneity. Ravits and La Spada^12^ hypothesized that despite disease heterogeneity, the disease poses common themes that may involve common mechanisms. They propose that ALS may in fact be an orderly, actively propagating process and that fundamental molecular mechanisms may be uniform.

The zebrafish is emerging as a useful tool for studying neurological diseases relevant to humans. Previously, we had shown that mutant *sod1* transgenic fish show the hallmarks of adult onset neurodegenerative ALS, including defective motor performance, motor neuron loss, a loss of neuromuscular connectivity, and muscle atrophy.^13^ The aforementioned observations demonstrate the usefulness of the zebrafish as a model for this disease.

However, among the current limitations when working with *in vivo* models of ALS is the lack of a good readout for the presymptomatic course of the disease. The zebrafish offer great advantages in studying early disease processes, as they develop rapidly, reaching postembryonic life at around 3 days postfertilization (dpf), which is developmentally similar to the neonatal mouse (for a comparison of developmental stages in human, mouse, and zebrafish, see Table 1). Moreover, the embryonic and larval zebrafish spinal cord is functionally and anatomically similar to that of humans, yet it is also optically transparent and experimentally accessible, making it ideal for the study of spinal circuits in normal and pathophysiological conditions.^14^

**TABLE 1:** Comparison of Neural Developmental Stages in Humans, Mice, and Zebrafish

	Embryo		Fetus	
	Neural groove	Neural tube	Spontaneous limb movement	Free living
Human	23 dpf	4 wpf	9-10 wpf	40 wpf

Mouse	8.5 dpf	9.5 dpf	12 dpf	21 dpf

	**Embryo**			**Larvae**
	**Neural thickening**	**Neural keel**	**Spontaneous movement**	**Free living**
Zebrafish	10.3 hpf	11.6-16 hpf	17-24 hpf	56-72 hpf

dpf: days post-fertilization; hpf: hours post-fertilization; wpf: weeks post-fertilization; dpp: days post partum. Larval zebrafish: 72hpf-30dpf; juvenile zebrafish: 30 dpf-3months; breeding adult: 3 months-2 years.

In the current study, we monitored *in vivo* early neurological changes caused by mutant *sod1* gene. The *sod1* zebrafish ALS model harbors a fluorescent heat shock stress response (HSR) reporter gene (*hsp70*-DsRed). The HSR is an endogenous cellular pathway that attempts to refold the damaged proteins in stressed cells, although this response is not always sufficient or beneficial.^15^ Thus, the HSR-mediated DsRed fluorescence in the *sod1* zebrafish model of ALS represents a useful tool for monitoring perturbations in cellular homeostasis caused by *sod1* mutation. This facilitates the mapping of disease focality and spread through the central nervous system (CNS) by the spatiotemporal readout of the neuronal stress response in the spinal cord of mutant zebrafish and provides an understanding of the cells and networks involved in disease propagation in ALS.

We present evidence that the HSR is an indicator of early pathogenic processes occurring in neurons. The HSR is first observed at embryonic stages, in discrete populations of inhibitory interneurons in the spinal cord, and is followed by dysregulation of glycine release from these inhibitory interneurons. Furthermore, we observe that following interneuron dysfunction, motor neurons start exhibiting neuronal stress. More interestingly, we show that motor neurons showing the HSR also show dysfunctional neuromuscular junctions (NMJs). Taken together, our observations suggest that the mutant sod1-induced HSR is a robust predictor of neuronal dysfunction and thus is a reliable marker of disease pathogenesis. Finally, we also show that the neuronal stress readout can be used to identify neuroprotective compounds such as riluzole and identify biological targets that may ameliorate early pathophysiological disease processes that are currently not well explored. Although the *sod1* zebrafish model may by itself not be sufficient in developing new therapies for ALS, this model system would provide a rapid way to triage compounds for screening in higher vertebrate models, with the potential for more rapid identification of promising compounds for translation into human clinical trials.

## Materials and Methods

Details are given in the Supplementary Materials and Methods.

### Generation of Transgenic Zebrafish

The zebrafish *sod1* transgenic fish lines were created according to protocols described previously by Ramesh et al.^13^ For all the transgenic strains, a suffix Sh was added (to imply Sheffield strain). The transgenic lines utilized for this study included the Tg(*sod1:sod1*^WT^;*hsp70:DsRed*)os4-Sh4, the line expressing the highest level of WTSod1 (×3.3 as compared to nontransgenic lines), referred to as WTos4-Sh4 line; Tg(*sod1:sod1*^G93R^; *hsp70:DsRed*)os10-Sh1, referred to as G93Ros10-Sh1 (high expresser with Sod1 expression increased ×3 and comparable to WTos4-Sh4); Tg(*sod1:sod1*^G93R^;*hsp70:DsRed*)os10-Sh2, referred to as G93Ros6-Sh2 (moderate expresser with Sod1 expression increased ×2.5); and Tg(*sod1:sod1*^G85R^; *hsp70:DsRed*) os6-Sh3 line, referred to as G85Ros6-Sh3 (low expresser with Sod1 expression increased ×1.5). When both G93R and G85R lines are discussed, they are referred to as MUT*sod1* lines.

### Electrophysiology

Whole cell voltage clamp recordings were conducted in 4 dpf larvae as previously described.^16^ The fish were perfused with Evans physiological saline containing the neuromuscular blocker D-tubocurarine (10μM), the sodium channel blocker tetrodotoxin (TTX; to synaptically isolate neurons), kynurenic acid (2.5mM, to block spontaneous glutamatergic currents), and bicuculline (25μM, to block spontaneous γ-aminobutyric acidergic [GABAergic] currents). Cells were voltage clamped at −75mV, a potential at which the chloride-conducting glycine receptors generate inward currents. Sulforhodamine (0.1%) was included in the electrode solution to visually identify the cell type. The frequency of glycinergic miniature postsynaptic currents (mPSCs) was determined by averaging the number of events in a 300-second period. To examine the rise time, decay, and amplitude of mPSCs, the first 50 mPSCs were selected from each recording and averaged across each experimental condition.

### Fluorescent RNA In Situ Hybridization

Fluorescent RNA *in situ* hybridization was performed on 24 hours postfertilization (hpf) G93Ros10 embryos, as previously described.^17^ A mix of equal concentrations of the probes (glyt2a, glyt2b, and DsRed; gad65,67 and DsRed; or vglut2 and DsRed) was used as previously described.^18^ Quantification of cells was done by counting DsRed/glycine-, DsRed/GABA-, and DsRed/vglut2-positive cells in the midtrunk region, and the percentage of DsRed cells showing glycine, GABA, or glutamate staining, as well as the percentage of each cell type showing DsRed staining, was calculated from an average of 10 embryos (a total of 429, 505, and 634 DsRed cells were counted, respectively, for each riboprobe pair).

### Immunofluorescence

Immunofluorescence was performed as described by Ramesh et al.^13^ Quantitative analysis of confocal images was performed on image stacks of 16 to 20μm thickness (0.5–1μm/section).

### Drug testing

The G93Ros10-Sh4 line was used to identify drugs that inhibit neuronal stress. Twenty-four hours postfertilization transgenic embryos (25 embryos/treatment) were incubated with sterile embryo media with the optimized concentration of the test compound and changed daily and maintained for 5 days. At 5 dpf, the lysates from transgenic or nontransgenic embryos were obtained by sonication (Sonicator 4000; Misonix, Farmingdale, NY) on ice followed by centrifugation. Fifty microliters of supernatant was analyzed in a 96-well plate (Corning-3880; Corning Life Sciences, Corning, NY) at DsRed wavelength.

### Image Analysis

Image analysis for NMJ analysis was performed using National Institutes of Health ImageJ software, and quantitative analysis of the NMJ morphology was performed using a colocalization analysis plugin.^19^,^20^

### Statistical Analysis

Statistics were performed using Prism 5 (GraphPad Software, La Jolla, CA). Unpaired *t* tests or analysis of variance with post hoc Bonferroni testing were used to compare groups. Electrophysiological data were compared using the 2-way Kolmogorov–Smirnov test.

## Results

### Induction of Neuronal Stress in Transgenic *sod1* Zebrafish

When developing the *sod1* transgenic zebrafish, we hypothesized that misfolding of the Sod1 protein in vulnerable cell populations would cause cellular stress and activate the HSR, allowing identification of potentially dysfunctional neurons. The HSR responsive, *hsp70*-*DsRed* construct that is inserted adjacent to the s*od1* gene is driven by the *hsp70* minimal promoter (1.5-kilobase fragment), and is not driven by the adjacent *sod1* gene. The basal *hsp70* promoter will allow induction of the DsRed reporter only in the presence of heat shock or cellular stress. In the absence of stress or heat shock, the promoter is silent, and no DsRed is synthesized. This method of reporter expression is commonly used in zebrafish research, and multiple lines within our lab using other transgenes (eg, *SMN*) made with this linked expression construct behave similarly.^21^

**FIGURE 1:**
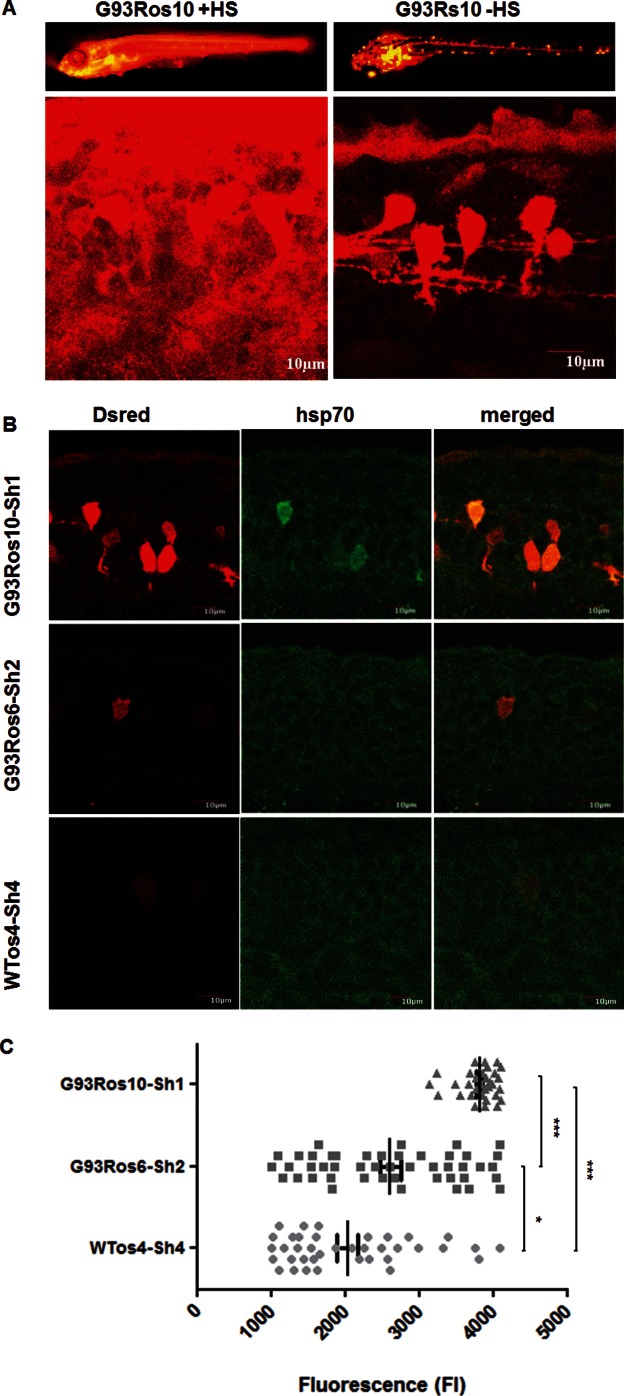
*Sod1* transgenic fish induce the heat shock response (HSR) without exposure to heat stress. (A) Live G93Ros10-Sh1 showing induction of *hsp70* measured by DsRed fluorescence at 7 days postfertilization in whole embryos (top panel) and 30 hours postfertilization (hpf) spinal cord (bottom panel). Larvae were heat shocked (+HS) or left unexposed to heat shock (−HS), and images of HSR induction were compared. When exposed to heat, the larvae showed global induction of the HSR (left panel), whereas in the absence of heat shock, only neuronal, neuroepithelial, and occasionally muscle cells show induction of the HSR (right panel). (B) Multiple mutant lines G93Ros10-Sh1 and G93Ros6-Sh2 show HSR induction in the absence of heat shock. Confocal images of spinal neurons show induction of endogenous hsp70 (middle column) in high expresser G93Ros10 line (top panel) in the same cells that showed strong DsRed expression (top row, left column). Moderate expresser G93Ros6-Sh2 line (middle row) and high expresser WTos4-Sh4 line (bottom row) do not show elevated hsp70 levels above background. (C) Quantitation of the DsRed fluorescence in individual neurons in the zebrafish embryonic spinal cord (30 hpf) by average fluorescence intensity. Average fluorescence of individual DsRed-positive neurons was measured and analyzed by analysis of variance. *p < 0.05 for G93Ros6-Sh2 and WTos4-Sh4; ***p < 0.0001 for G93Ros10-Sh1 and WTos4-Sh4. Size bars = 10μM. [Color figure can be viewed in the online issue, which is available at http://www.annalsofneurology.org.]

We generated multiple G93R (high expresser: G93Ros10-Sh1; moderate expresser: G93Ros6-Sh2), G85R (low expresser: G85Ros6-Sh3), and wild-type (WT; high expresser: WTos4-Sh4) *sod1* transgenic zebrafish carrying the *hsp70-DsRed* stress reporter gene. As expected, the *sod1* transgenic fish showed red fluorescence throughout the body and also in the spinal cord upon heat shock (Fig 1A, +HS). Interestingly, we found that in the absence of heat shock the embryos expressing Sod1 exhibited specific DsRed expression in the CNS, indicating the presence of neuronal stress (see Fig 1A,−HS). The high (G93Ros10-Sh1) and moderate expresser (G93Ros6-Sh2) mutant *sod1* lines showed DsRed induction in distinct neuronal groups in the dorsal spinal cord. The highest expresser WTos4-Sh4 line showed a minimal level of DsRed induction, despite having high transgene copy numbers and 3-fold increased Sod1 protein expression; levels were identical to the G93Ros10-Sh1 mutant line. Most importantly, endogenous hsp70 was also upregulated in cells that expressed DsRed, evident in the neurons that show high DsRed expression levels. The moderate expresser mutant and the high expresser WT lines did not show strong DsRed or detectable hsp70 induction. DsRed is a very stable protein, and this stability greatly amplifies the hsp70 induction signal. This was shown by a strong DsRed signal persisting for several days after heat shock (Supplementary Fig 1E, F) in the transgenic fish, whereas endogenous hsp70, which is tightly regulated,^22^ returned to background levels at 15 hours after heat shock.

### Stressed Neurons in the Embryonic Spinal Cord Are Predominantly Inhibitory Glycinergic Interneurons

The zebrafish spinal cord is composed of neurons and glia.^23^ The neurons include a variety of interneurons and motor neurons, whereas the glial population consists of oligodendrocytes that myelinate axons and radial glial cells (similar to astrocytes) that provide support to neurons.^23^ To determine the spatiotemporal onset of the HSR in the spinal cord, we used *in situ* hybridization and antibody staining to determine the identity of DsRed-expressing cells in mutant embryos and larvae. Interestingly, DsRed expression was never observed in oligodendrocytes, radial glia, or motor neurons (*olig2-* or *hb9*-positive early differentiating and *ChAT*-positive mature) in the mutant *sod1* larvae (Supplementary Fig 2).

**FIGURE 2:**
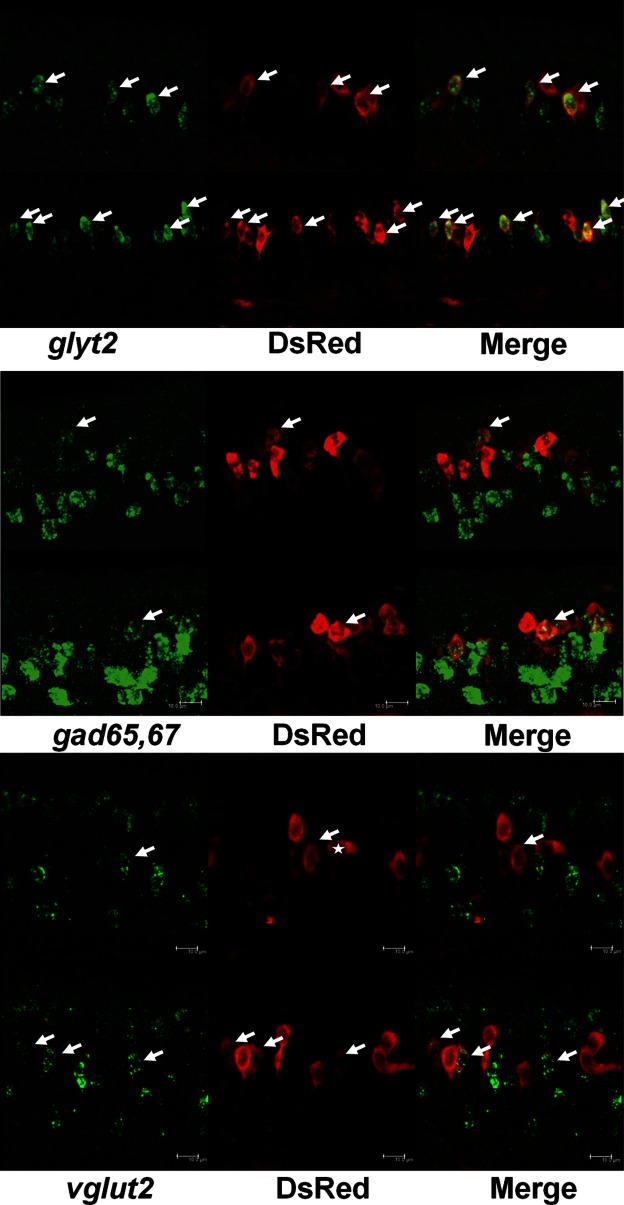
Embryonic mutant *sod1* zebrafish show induction of neuronal stress predominantly in the spinal inhibitory glycinergic interneurons. *In situ* hybridization with probes for inhibitory and excitatory neurons (left panels) and DsRed riboprobes (middle panel). Top panel: glyt2-positive glycinergic inhibitory interneurons; middle panel: gad65,67-positive γ-aminobutyric acidergic inhibitory interneurons (dorsal longitudinal ascending and some glycinergic interneurons); bottom panel: vglut2-positive excitatory interneurons (commissural primary ascending and commissural secondary ascending interneurons) that cross-modulate the spinal locomotor circuitry. Time: 24 hours postfertilization. Size bars = 10μM. [Color figure can be viewed in theonline issue, which is available at http://www.annalsofneurology.org.]

Thus, we reasoned that the first spinal neurons to activate HSR were likely to be interneurons. We next sought to determine which interneuron populations exhibit the HSR. The zebrafish spinal cord has been characterized in detail, and the different spinal interneurons have stereotyped neuroanatomy, with predictable anatomical positions and axonal trajectories.^24^ Moreover, each class expresses 1 of only 3 neurotransmitters: glycine, GABA, and glutamate.^24^ We therefore used a combination of anatomical and transmitter expression characteristics to identify the cell types in which the HSR was present.

To determine whether the stressed neurons of *sod1* mutant embryos were glycinergic, we used dual color fluorescence *in situ* hybridization with riboprobes targeted against glycine transporter (*glyt2a, b*) and *DsRed* RNA. We observed numerous cells in which *glyt2a,b* and DsRed colocalized (Fig 2, top panel). Similarly, DsRed expression often colocalized with cells positive for antiglycine antibodies (Supplementary Fig 3B). Quantitation of the percentage of glycinergic interneurons that showed the neuronal stress response revealed that almost half of the glycinergic interneurons (49.23 ± 12.8 percent) showed DsRed expression at 24 hpf. The percentage of DsRed-positive neurons that were glycinergic was 44.2 ± 9.5%.

**FIGURE 3:**
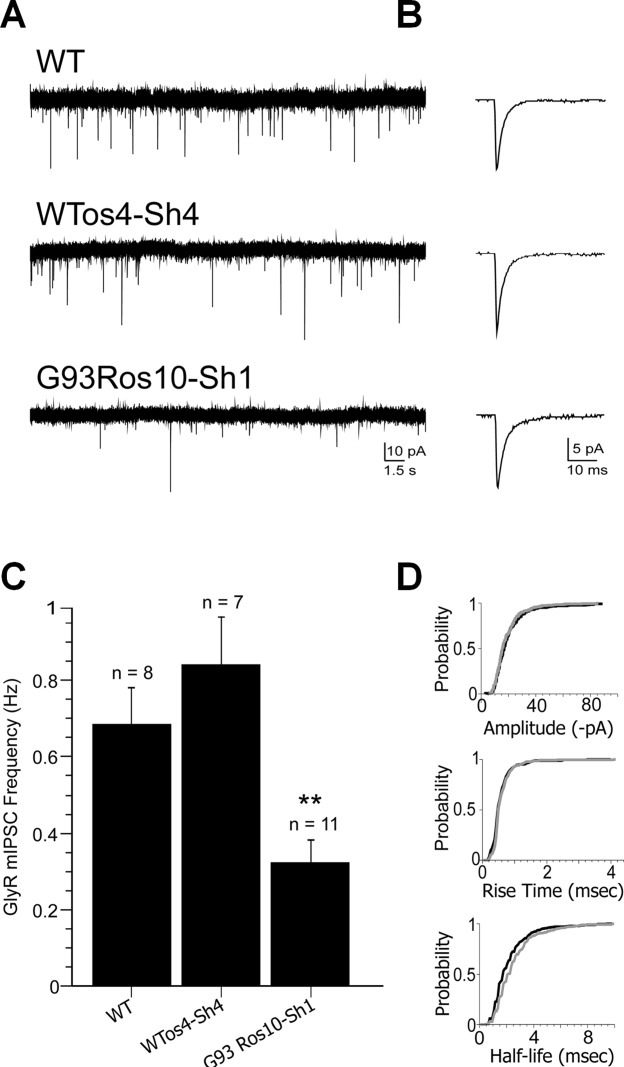
Reduced glycinergic transmission onto motor neurons of *sod1* zebrafish larvae. (A) Representative traces depicting voltage clamp (holding potential = −75mV) recordings of spontaneous glycinergic miniature postsynaptic currents (mPSCs) in motor neurons of wild-type (WT), WT Sod1 overexpresser (WTos4-Sh4), and *sod1* mutant (G93Ros10-Sh1) fish at 4 days postfertilization. Downward deflections represent occasional quantal release of glycine from presynaptic terminals. (B) Average of 30 consecutive glycinergic mPSCs from each experimental condition. (C) Bar chart depicting mean mPSC frequency for each experimental condition. GlyR = glycine receptor; mIPSC = miniature inhibitory postsynaptic current. **WT vs G93R *p* < 0.001. (D) Cumulative probability plots of mPSC amplitude, rise time, and half-life (*p* < 0.05) in WT (*black lines)* and G93Ros10-Sh1 (*gray lines)* motor neurons.

The majority of DsRed-positive cells that coexpressed glycine had dorsally located perikarya and axons that projected rostrally through the dorsolateral aspect of the spinal cord toward the brain. Such axonal projections are observed in commissural secondary ascending (CoSA)–glycinergic (mammalian V0-like), commissural bifurcating longitudinal (CoBL; mammalian dl6-like), and circumferential ascending (mammalian V1-like) interneurons, which comprise the 3 glycinergic interneurons of the zebrafish spinal cord at this stage of development.^24^,^25^

Of these, the CoBL and CoSA interneurons are pax2 positive.^18^,^25^,^26^ We therefore used anti-pax2 antibodies to label CoBL and CoSA interneurons. However, as our DsRed and pax2 antibodies were both rabbit derived, we were limited to monitoring pax2 colocalization within strongly DsRed-positive cells unenhanced by immunolabeling. Multiple fields with interneurons that showed strong DsRed expression showed colocalization with pax2 antibody staining (see Supplementary Fig 3A).

To determine whether GABAergic interneurons expressed the HSR, we performed *in situ* hybridization with riboprobes targeted against GABA biosynthetic enzymes gad65 and gad67. We found that 14.2 ± 6.8% of DsRed-positive neurons had a GABAergic transmitter phenotype (see Fig 2, middle panel), whereas the percentage of GABA-positive neurons that showed HSR was 8.18 ± 4.95%.

We next asked whether the HSR occurred in the glutamatergic interneurons of the mutant embryonic spinal cord. Investigation of the expression of the glutamatergic excitatory neurotransmitter using vglut2 riboprobes showed that 23 ± 5.4% of the DsRed-positive neurons were vglut2 positive (see Fig 2, bottom panel). Analysis of the percentage of vglut2-positive neurons that showed the HSR indicated that 10.8 ± 1.94% of vglut2 neurons were DsRed positive.

Glutamatergic interneurons in zebrafish have primary ascending or descending axons. Among them, the commissural primary ascending (CoPA) and CoSA-glutamatergic interneuronal subtypes have ascending axons.^24^ After careful examination of >25 DsRed-labeled embryos, we were unable to see any interneurons with primary descending axons. Hence, we believe that the vglut2-positive interneurons that show the HSR are either CoPA or CoSA-glutamatergic interneurons. Thus, the populations of neurons that show the HSR in the embryonic spinal cord were primarily inhibitory glycinergic interneurons, with some GABAergic and glutamatergic neurons that modulate the local reciprocal activation and inhibitory circuits required for swimming.

### Reduced Glycinergic Input onto Motor Neurons following Induction of Interneuron Stress

To this point, our findings suggested that glycinergic inhibitory interneurons comprise the majority of stressed neurons in the early mutant *sod1* zebrafish spinal cord. Hence, we used *in vivo* patch clamp electrophysiology to monitor glycinergic inputs onto motor neurons at 2 dpf, a time when the stress response is just manifesting, and at 4 dpf, a time when the stress response is pervasive. Glycinergic transmission was monitored during voltage clamp recordings by synaptically isolating neurons with the sodium channel blocker TTX and blocking glutamate and GABA transmission with kynurenic acid (2.5mM) and bicuculline (25μM), respectively. Under these conditions, spontaneous mPSCs were observed in motor neurons that represented quantal release of glycine from glycinergic interneurons (Fig 3A). The frequency of these events did not differ significantly in nontransgenic and WTos4-Sh4 fish (control = 0.68 ± 0.09Hz, WTos4-Sh4 = 0.84 ± 0.13Hz; *p* > 0.05) but was approximately 50% lower in motor neurons of *sod1* mutant fish (0.32 ± 0.05Hz; *p* < 0.001; see Fig 3A, C). In addition, cumulative probability plots revealed a 15% increase in the half-life (control = 2.21 ± 0.08 milliseconds vs *sod1* mutant = 2.55 ± 0.08 milliseconds; *p* < 0.05), but no change in rise time (control = 0.58 ± 0.02 milliseconds vs *sod1* mutant = 0.60 ± 0.04 milliseconds; *p* > 0.05) or amplitude (control = 20.27 ± 0.63pA vs *sod1* mutant = 18.9 ± 0.5pA; *p* > 0.05) of mPSCs (see Fig 3D). However, at 2 dpf, there were no differences in these parameters between the WT and mutants (data not shown). Taken together, these data indicate that the stress response in glycinergic interneurons is predictive of their impaired function.

### Chronic Loss of Inhibitory Input May Contribute to Motor Neuron Stress

As the motor neurons show dysregulation of inhibitory input within a few days after birth, potentially due to pathophysiological changes within inhibitory interneurons, we hypothesized that loss of inhibitory input may contribute to the onset of motor neuron stress, due to chronic loss of inhibitory interneuron input.

We had earlier shown that staining for pre- and postsynaptic NMJ markers was reduced in the muscles of 11dpf *sod1* transgenic zebrafish.^13^ However, at these larval stages NMJs were not grossly perturbed, indicating that motor neuron loss was unlikely to have occurred at the stage when DsRed expression is first observed in the interneuron population.^13^ Analysis of DsRed expression in the high expresser G93Ros10-Sh1 line in 9 dpf larvae (which represent a similar developmental stage to the preweaning stages in mice) showed widespread stress in interneurons, but an absence of stress in motor neurons, suggesting that motor neurons do not show the HSR at the early larval stage of development (Supplementary Fig 4).

**FIGURE 4:**
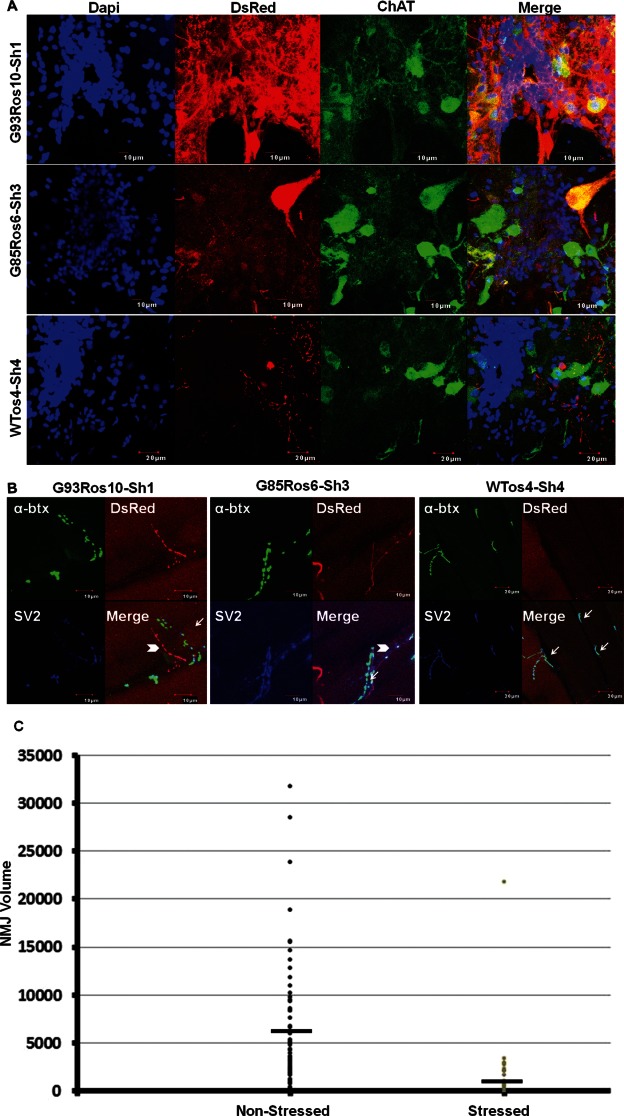
Mutant *sod1* zebrafish show stress in the large spinal motor neurons of the adult spinal cord and concurrent loss of neuromuscular junction (NMJ). (A) Spinal cord cross sections from 1- to 1.5-year-old adult zebrafish stained with 4′,6-diamidino-2-phenylindole (Dapi), DsRed antibody, and ChAT antibody show robust induction of the heat shock stress response in spinal cord motor neurons. DsRed colocalized with ChAT in the high expresser (×3) G93Ros10-Sh1 line (top panel) and the moderate expresser (×2) G85Ros6-Sh3 line (middle panel). The high expresser (×3) WTos4-Sh4 line shows little DsRed expression, and DsRed did not colocalize with the large ChAT-positive motor neurons (bottom panel). (B) Muscle sections labeled with synaptic vesicle-2 (SV2) antibody (blue), α-bungarotoxin(α-btx) (green), and DsRed (red) in high expresser G93Ros10-Sh1 (left panel), low expresser G85Ros6-Sh3 (middle panel), and high expresser WTos4-Sh4 (right panel). Normal NMJs are indicated by arrows. Abnormal NMJs (*arrowheads)* where pre- and postsynaptic markers are absent or small and punctate were detected in the muscle sections from the mutant lines (left and middle panels) but not in the high expresser wild-type line (right panel). (C) One hundred thirteen NMJs from multiple sections were measured for NMJ volume from confocal stacks across multiple planes in SV2-positive–DsRed-negative axons and SV2-positive–DsRed-positive axons using colocalization software from National Institutes of Health Image J and analyzed by unpaired *t* test. Significant reduction in NMJ volume was observed associated with stressed motor axons as compared to the nonstressed axons. The mean is represented as a line over the distribution. Each dot represents the volume of an individual NMJ. *p* < 0.00001. [Color figure can be viewed in the online issue, which is available at http://www.annalsofneurology.org.]

We examined whether symptomatic adult zebrafish, which were 12 months old, show evidence of motor neuron stress. Interestingly, spinal motor neurons from mutant *sod1* adult fish aged 12 to 18 months, which exhibit reduced motor function, showed evidence of motor neuron stress (Fig 4A, top and middle panels). Greater DsRed induction in the spinal cord of the high expresser transgenic line as compared to the low expresser was observed. However, unlike the 3 independent mutant *sod1* lines, no DsRed expression was observed in the spinal motor neurons of the high expresser WTos4-Sh4 line (see Fig 4A, bottom panel). This mutant *sod1*-specific HSR could also be observed in the motor neurons of young adult zebrafish at 6 months of age (Supplementary Fig 5). Thus, it appears that the HSR in motor neurons is induced between 9 dpf and 6 months of age.

**FIGURE 5:**
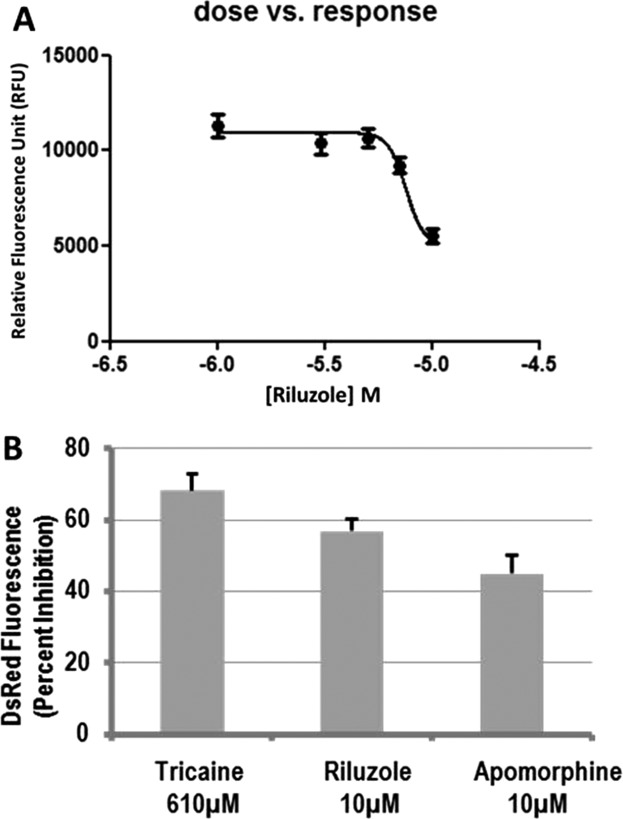
Inhibition of the stress response in *sod1* G93Ros10-Sh1 zebrafish embryos by riluzole and NRF2 activator R-apomorphine. (A) Dose–response curve showing dose-dependent inhibition of the stress response by riluzole in *sod1* G93Ros10-Sh1 embryos treated for 4 days with 1, 3, 5, 7, and 10μM riluzole, *p* < 0.001. (B) Percentage inhibition of the stress response expressed by reduction in DsRed fluorescence in embryos treated with 610μM Tricaine (*p* < 0.000001), 10μM riluzole (*p* < 0.00001), and 10μM R-apomorphine (*p* < 0.001) as compared to 0.1% dimethyl sulfoxide–treated embryos. Mean ± standard error of the mean.

### Stressed Motor Neurons Show Reduced Neuromuscular Synaptic Volume and Loss of Muscle Innervation

How muscle denervation occurs in ALS is a fundamental question that remains unanswered, and conflicting data indicate that both muscle and motor neurons play an important role in disease pathogenesis.^27^–^29^ We had previously shown that, similar to both mice and humans, the number of NMJs in the G93Ros10 mutants was reduced, and the few that remained were abnormally small and punctate in appearance.^13^ However, it was unclear as to whether these perturbations occurred only at sites innervated by stressed motor neurons. To determine whether this was the case, we compared NMJs innervated by stressed (DsRed-positive) and nonstressed (DsRed-negative) motor neurons. DsRed-positive axons were detected in the musculature of the mutant (see Fig 4B, left and middle DsRed panels) but not in the WT *sod1* transgenic zebrafish (see Fig 4B, right DsRed panel).

We also observed that the DsRed-positive axons in the high expresser G93Ros10-Sh1 showed no identifiable NMJ structures and were completely devoid of α-bungarotoxin–labeled postsynaptic structures (see Fig 4B, left α-btx panel). However, in the low expresser G85Ros6-Sh3 muscle sections, we observed that the DsRed-positive axons showed some recognizable NMJs, which were, however, abnormally small in size and often punctate in appearance (see Fig 4B, middle α-btx panel). In contrast, the nonstressed axons in the same section that did not show DsRed expression showed large, well-developed NMJs (see Fig 4B, left and middle α-btx panels).

The high expresser G93Ros10-Sh1 line showed almost no NMJs arising from the DsRed-expressing stressed axons, thus limiting quantitative analysis of the NMJ in this line. Hence, we chose to use the low expresser G85Ros6-Sh3 line for quantitation of NMJ volume in axons derived from stressed and unstressed neurons. The G85Ros6-Sh3 line showed some NMJ structures associated with the stressed axons and allowed appropriate measurement of the effects of stress within the axonal compartment on NMJ integrity. NMJs were identified at the distal ends of stressed and nonstressed axons, and the NMJ volume within the region of interest was quantitated using 3-dimensional images from confocal stacks.^19^,^20^ We observed an almost 5.5-fold decrease in NMJ volume associated with stressed axons as compared to nonstressed axons (1,097 ± 477 vs 6,245 ± 864; *p* < 0.00001; see Fig 4C). The NMJs in the *WTsod1* transgenic line were normal and showed large and well-developed synapses (see Fig 4B, right α-btx panel).

### Riluzole and NRF2 Activators Reduce Neuronal Stress

The major impetus for developing the zebrafish ALS model is their suitability for high-throughput drug screening, thereby facilitating discovery of drugs that ameliorate ALS. To determine whether the zebrafish model has the potential to identify novel ALS therapies, we tested the ability of the antiexcitotoxic drug riluzole to modify neuronal stress in zebrafish larvae. Riluzole was chosen for this study as it is the only drug shown to have a disease-modifying effect in ALS patients. We subjected 24 hpf G93Ros10-Sh1 embryos to a 4-day incubation in 1, 3, 5, 7, and 10μM riluzole and used DsRed fluorescence as a marker for progressive changes in the neuronal stress response. We observed that riluzole caused a dose-dependent reduction in DsRed fluorescence, with an IC_50_ of approximately 7μM (Fig 5). Tricaine, a local anesthetic that inhibits neuronal sodium channels^30^ like riluzole and also reduces excitotoxicity, produced a significant decrease in DsRed fluorescence (see Fig 5B).

The NRF2 (nuclear factor [erythroid-derived 2]-like 2) pathway plays an important role in regulating oxidative stress, the cellular handling of misfolded proteins, and mounting the autophagy response.^31^,^32^ Thus, we hypothesized that drugs that upregulate this pathway may potentially reduce neuronal stress in this model system. We used R-apomorphine, which is known to be an activator of the NRF2 antioxidant response (ARE)^33^ to determine the effects of ARE induction on the stress response observed in this model system. Treatment of embryos with R-apomorphine produced a significant reduction in the neuronal stress response (see Fig 5B). Thus, 2 neuroprotective drugs acting through diverse mechanisms were able to reduce the readout of neuronal stress in this model system.

## Discussion

Zebrafish have become a powerful model for the study of degenerative diseases, as their experimental accessibility, small size, and genetic similarity to mammals facilitates detailed analysis of disease mechanisms and drug screens. Zebrafish expressing mutant sod1 develop hallmark features of ALS commonly associated with both murine models and the human disease^13^ (a comparison of pathophysiological changes in our *sod1* zebrafish model, *SOD1* mouse models, and human ALS is summarized in Table 2).

**TABLE 2:** Summary of ALS Disease Pathophysiology in Humans, Mice, and Zebrafish

	Neuronal stress	Electro physiology	NMJ denervation	Cellular changes, insoluble aggregates	Astrocytosis/ Microgliosis/ Mitochondria/ Inclusion/UPS	Gross motor symptoms
Human	?	Prior to onset in adults†	Prior to onset in adults†	Golgi, mitochondria, insoluble aggregates In post-mortem specimens	In post-mortem specimens	40-50 years

Mouse (Sod1G93 AHiGur)	?	12 dpf* to 6 dpp** (G85R, G93Alow)	∼30dpp	Golgi changes ∼40-50dpp	∼70-90dpp	∼100dpp

Zebrafish (G93R)	24 hpf ** (This manuscript)	96hpf** (This manuscript)	Normal NMJ but reduced staining at 11dpf. 6 months and 1 year Observed denervation in stressed motor neuron (This manuscript)	UPR at 1 month***.	No gross inflammation (unpublished) Motor neuron stress. 6-months (This manuscript)	1 year

dpf: days post fertilization; hpf: hours post fertilization; dpp: days postpartum. UPR: Unfolded Protein Response †earliest tested, * earliest tested in organotypic culture; ** Earliest tested in intact spinal cord, *** Personal communication (C.Beattie)

In the current study, we have extended our analysis of mutant zebrafish to track early, presymptomatic perturbations associated with the Sod1 mutation. Using the *hsp70-DsRed* marker gene as a novel readout of neuronal stress, we have characterized perturbations in the spinal cord from early embryonic stages through to adult life, establishing a correlation between neuronal stress and pathophysiological changes in stressed neurons. We find that in early stages of development, inhibitory neurons are sensitive to the presence of mutant Sod1, activating the stress response upon its expression. Moreover, using electrophysiological approaches, we show that glycinergic neurotransmission onto motor neurons is impaired in mutant *sod1* fish. These perturbations precede the onset of pathophysiological defects in the motor neurons and at the neuromuscular junction, which occur later in life.

SOD1 is a ubiquitously expressed protein, but mutation in the *SOD1* gene produces a disease affecting the CNS. How this gene selectively perturbs neurons during ALS is not well understood, although the accepted view is that neurons are selectively sensitive to mutant SOD1. Our observation suggests that neuronal stress as measured by the HSR response occurs in spinal interneurons long before it is observed in motor neurons. This implicates interneurons as important components of ALS disease progression in this mutant *sod1* zebrafish model. More importantly, we provide for the first time evidence that this process occurs *in vivo*.

A possible role for inhibitory interneurons in ALS has been hypothesized previously (see Turner and Kiernan^34^ for a comprehensive review on this subject). Both human ALS patients and mouse *SOD1* models have been reported to show a loss of spinal cord interneurons, aberrant recurrent inhibition, and motor neuron hyperexcitability, observations that suggest that dysregulation of inhibitory influences on motor neurons may represent an early aspect of disease pathophysiology.^11^,^35^–^38^ Moreover, recent *in vitro* studies show that motor neurons derived from *SOD1* mice have abnormal glycine receptor expression and reduced responses to glycinergic input.^39^ As shown in Table 2, electrophysiological abnormalities are the earliest changes observed in mice, suggesting that early pre- and postnatal development is an area important for investigation in relation to the mechanisms of SOD1 toxicity. Together, these observations suggest that defective inhibitory transmission may promote motor neuron stress and accelerate disease progression. It is perhaps worth noting that that human patients carrying the SOD1D90A homozygous mutations develop an atypical slowly progressing form of ALS, and in these patients, inhibitory interneurons are spared.^40^ However, Hossaini et al^41^ reported that interneuron pathology occurred following motor neuron death in the low expresser SOD1G93A mice. This study was limited to examining adult mice, a few weeks prior to the onset of symptoms, and did not look at early embryonic or neonatal animals. Interestingly, mice from this line show evidence of hyperexcitability as early as postnatal day 6 to 10, long before the onset of symptoms.^42^ The source of this hyperexcitability is still unclear, but the data presented in this paper raise the possibility that dysfunctional inhibitory interneurons may be responsible for these changes. Future studies looking at different neuronal populations during embryonic, neonatal, and adult stages of development in the mutant *SOD1* mouse model would be necessary to establish the exact timing of damage to various cell types.

Interestingly, overexpression of WT Sod1 also induced neuronal stress in interneurons, albeit at a much lower level than in the mutant *sod1* zebrafish lines. This observation stands in agreement with previous studies that show high levels of WT SOD1 are toxic, although to a much lesser extent than mutant SOD1.^3^,^5^,^43^ For example, transgenic mice that overexpress WT SOD1 also show motor neuron loss, although it occurs far later (2 years).^44^ It is important to note that the high expresser G93Ros10 line that expresses similar levels of Sod1 to the WTos4 line showed a 10-fold greater HSR induction compared to the WT-expressing line. Similar to the mouse overexpressing WTSOD1 where the disease was limited and not progressive, in WTSod1-overexpressing transgenic zebrafish, the HSR failed to spread to motor neurons, to produce NMJ denervation, or to cause muscle atrophy. Our observations are in keeping with those seen in WTSOD1-overexpressing transgenic mice. Another interesting finding from our study is that motor neurons did not show induction of the HSR at the early embryonic and larval stages, a time when inhibitory interneurons exhibited clear dysfunction. Rather, the onset of HSR induction in motor neurons occurred long after, as the fish progressed toward adult life. These findings suggest that the pathophysiological changes are not static but spread progressively through the neural network controlling motor system function. The findings in this zebrafish model indicate that inhibitory interneurons may be the cell type within the spinal cord most susceptible to neuronal stress induced by the presence of mutant Sod1. One route for the propagation of the pathophysiology from inhibitory interneurons to motor neurons is through dysregulation of the inhibitory input to motor neurons, as discussed earlier. The zebrafish model we have generated offers new approaches to test this hypothesis and identify the circuitry involved in the pathophysiological cascade.

Dying back axonal pathology is among the proposed mechanisms leading to motor neuron death, although we do not know whether this occurs as a primary event or secondary to changes in the motor neuron perikaryon.^45^,^46^ We had earlier shown that NMJs in mutant *sod1* transgenic strains were reduced, and the few that were present were morphologically abnormal (small and punctate). This HSR readout in our zebrafish model allows us to track the axons of stressed motor neurons into the muscle and evaluate whether stress in the motor neuron perikaryon leads to detectable pathology at the NMJ. We show that NMJs from stressed motor neurons were abnormal, as they were absent or were small and punctate in appearance as compared to nonstressed axons. This effect was specific to the mutant *Sod1* lines and was not observed in the WT Sod1 overexpressing line. This indicates that the stress we observe in the motor neuron perikarya is predictive of synaptic degeneration at the NMJ. The *sod1* zebrafish model will allow more detailed dissection of the processes involved in denervation at the NMJ, as now we can distinguish motor axons arising from nonstressed and stressed motor neurons.

**FIGURE 6:**
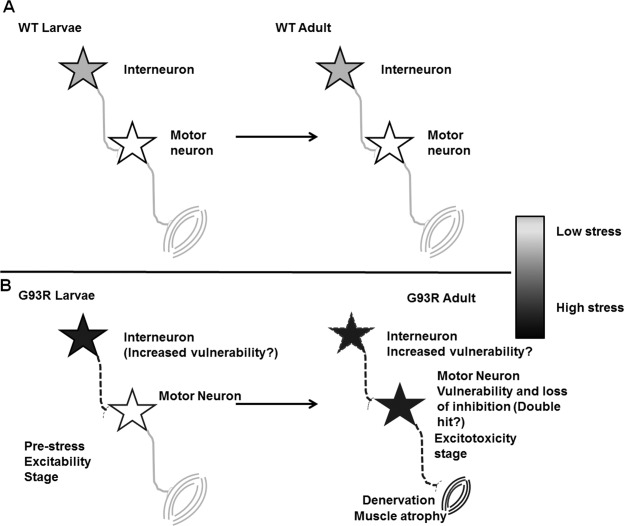
Model of neuronal stress propagation in zebrafish model of amyotrophic lateral sclerosis. (A) Although wild-type (WT) Sod1 overexpresser zebrafish show some interneuron stress in early development, the stress levels are low and there is no propagation of the stress response to motor neurons. (B) Mutant *sod1* transgenic zebrafish show stress initially in the inhibitory interneurons that cause dysfunction of glycinergic inhibitory interneurons with reduced glycinergic input to motor neurons. This lack of inhibition may be a factor contributing to the stress response developing in motor neurons in the adult zebrafish. Stressed motor neurons are unable to maintain synaptic function with resulting synaptic withdrawal at the neuromuscular junction (NMJ). The retraction of the presynaptic input at the NMJ may lead to a loss of trophic support from muscle, resulting in a vicious cycle of injury to the motor neuron, eventually leading to motor neuron degeneration and muscle atrophy. Shades of gray indicate stress levels.

Based on the combination of novel findings in the *sod1* zebrafish model of ALS, we propose a draft model of ALS disease propagation in which neuronal stress begins very early in life, initially affecting the inhibitory interneuron pool, whose dysfunction may then contribute to pathophysiological changes in motor neurons later in life (Fig 6B). In this proposed model, it is also possible that interneurons are more vulnerable to the toxic effects of mutant Sod1, whereas motor neurons may require 2 hits (Sod1 toxicity and loss of inhibition) to drive pathology. Future cell-specific transgene expression and/or tissue transplant studies, which can be performed in zebrafish embryos, will allow further exploration of this hypothesis. Nonetheless, stressed motor neurons are unable to maintain normal NMJs, which may in turn lead to lack of the trophic support necessary for maintenance of functional neuromuscular contacts, thereby leading to denervation. Such a model has been previously suggested,^46^ but here we propose that stressed motor neurons are dysfunctional and induce a dying back phenotype, whereas NMJs of nonstressed neurons remain intact. The evidence we provide here shows a direct link between the level of chronic neuronal stress and neuromuscular degeneration.

Although we note that the early disease features seen in this zebrafish model (electrophysiological changes and NMJ denervation) are also observed in the presymptomatic rodent models of SOD1-related ALS and to a limited extent in presymptomatic human patients, further work is necessary to determine whether these early changes reflect the initiation of a progressive pathophysiological cascade that eventually culminates in motor neuron loss. However, the similarities in the pathophysiological changes occurring across these diverse species and the gradual nature of these changes that precede motor neuron loss suggest that they are likely to represent an integral component of the disease process. Nevertheless, the ability to use zebrafish to monitor these changes in real time *in vivo* due to their small size, transparency, and easy accessibility provides us with a valuable tool for studying the cascade of motor neuron injury in ALS from early pathophysiological changes to motor neuron cell death.

The *sod1* zebrafish model also holds promise as a link between cell-based assays and rodent models, thus providing a new platform for high-throughput screening of neuroprotective compounds. In the current study, we tested the effects of riluzole, the only therapeutic compound that has shown benefit in both the SOD1^G93A^ murine models and human ALS patients, on neuronal stress in the mutant *sod1* zebrafish larvae. We found that riluzole reduced the HSR in zebrafish, indicating that it may slow progression of disease. Although riluzole shows efficacy in ALS, the mechanism by which it mediates neuroprotection is still unclear. Riluzole is known to reduce neuronal excitability by stabilizing the inactive state of voltage-gated sodium channels and also by acting as a noncompetitive N-methyl-d-aspartate receptor antagonist.^47^ Interestingly, Tricaine, another drug that modulates voltage-gated sodium channels, showed a similar inhibition of the HSR. Together, these observations suggest that modulation of neuronal sodium channel activation may affect disease progression, possibly in an activity-dependent manner. Dissecting the molecular mechanisms by which riluzole reduces the neuronal stress response will be useful and may allow the development of compounds with greater neuroprotective efficacy. We also identified that activators of the NRF2 transcription factor, which plays an important role in neuroprotection and operates as a master regulator of the antioxidant and stress response pathways, can also modulate the early neuronal stress response observed in this model. Although this early stage screening allowed identification of neuroprotective compounds, further validation of hits from this assay will be necessary in higher vertebrate models of ALS. The *in vivo* high throughput zebrafish model fills an important gap in ALS drug discovery and may provide lead candidates for drug screening in rodent models, thus accelerating the drug discovery process in ALS with the potential for more rapid identification of promising compounds for clinical translation.

## Supplementary material

Supporting Information Figure 1. DsRed provides a stable and cumulative readout of endogenous hsp70 induction: Whole-mount antibody labelling with hsp70 (green) (A-D) and DsRed (E,F) in non-heat shocked control (A,C,E) and heat shocked (B,D,F) zebrafish embryos at 29 hpf (A,B), 39 hpf (C,D) and 48 hpf (E,F). Magnification: 20X

Supporting Information Figure 2. Spinal motor neurons and glial cells do not show induction of the HSR in mutant sod1 transgenic spinal cord at the early larval stages. DsRed (middle panel) did not colocalise with GFP in Sod1-G93Ros10-Sh1 transgenic zebrafish crossed with hb9-GFP (row1), islet-1-GFP (row 2) (markers of differentiating motor neurons) and did not immunolabel with ChAT antibody (row 3)(marker of mature motor neurons). DsRed also did not colocalise with migrating GFP positive oligodendrocytes (arrowhead) in crosses with olig2-GFP fish (row4) or with GFP positive radial glia in crosses with GFAP-GFP zebrafish (row 5). Time 72 hpf. Scale bar 10μm.

Supporting Information Figure 3. DsRed colocalises with pax2 positive glycinergic interneurons: Whole-mount antibody labelling with anti-pax 2 antibody (green, Top panel) and DsRed (middle panels) and glycine antibody (green, bottom panel) in non-heat shocked G93Ros10-Sh1 embryos at 48 hpf. Neurons showing colocalization are marked by arrows. Scale bar 10 μm (top panels), 5μm (bottom panels)

Supporting Information Figure 4. Zebrafish larvae at 9 dpf do not show induction of stress response in motor neurons. Cross sections of 9 dpf spinal cord immunolabelled with ChAT (column 3, arrow and area in triangle) and DsRed (column 2). Cell nuclei are labelled by Dapi (column 1) and the merged image (column 4) shows no DsRed/ChAT positive motor neurons. DsRed positive neurons are dorsally located in the spinal cord. Scale bar 5μm (top panel), 10μ m (bottom panel)

Supporting Information Figure 5. Motor neuron stress is observed in 6 month and 1 year old spinal cords from mutant sod1 transgenic zebrafish. Cross sections of spinal cord from 6 month (top of each group) and 1 year (bottom of each group) old high expressor G93Ros10-Sh1 (top 2 panels) and low expressor G85Ros6-Sh3 (bottom 2 panels) transgenic zebrafish immunolabelled with ChAT (column 3) and DsRed (column 2). The cell nuclei are labelled with Dapi (column 1) and the merged image (column 4) shows DsRed/ChAT positive motor neurons (arrows). Magnification 40X.
