# Supplementary figures and images for: Early Interneuron Dysfunction in ALS: Insights from a Mutant sod1 Zebrafish Model
Source: Ann Neurol. 2012 Dec 31;73(2):246–58. doi: 10.1002/ana.23780 (PMC3608830; doi:10.1002/ana.23780)

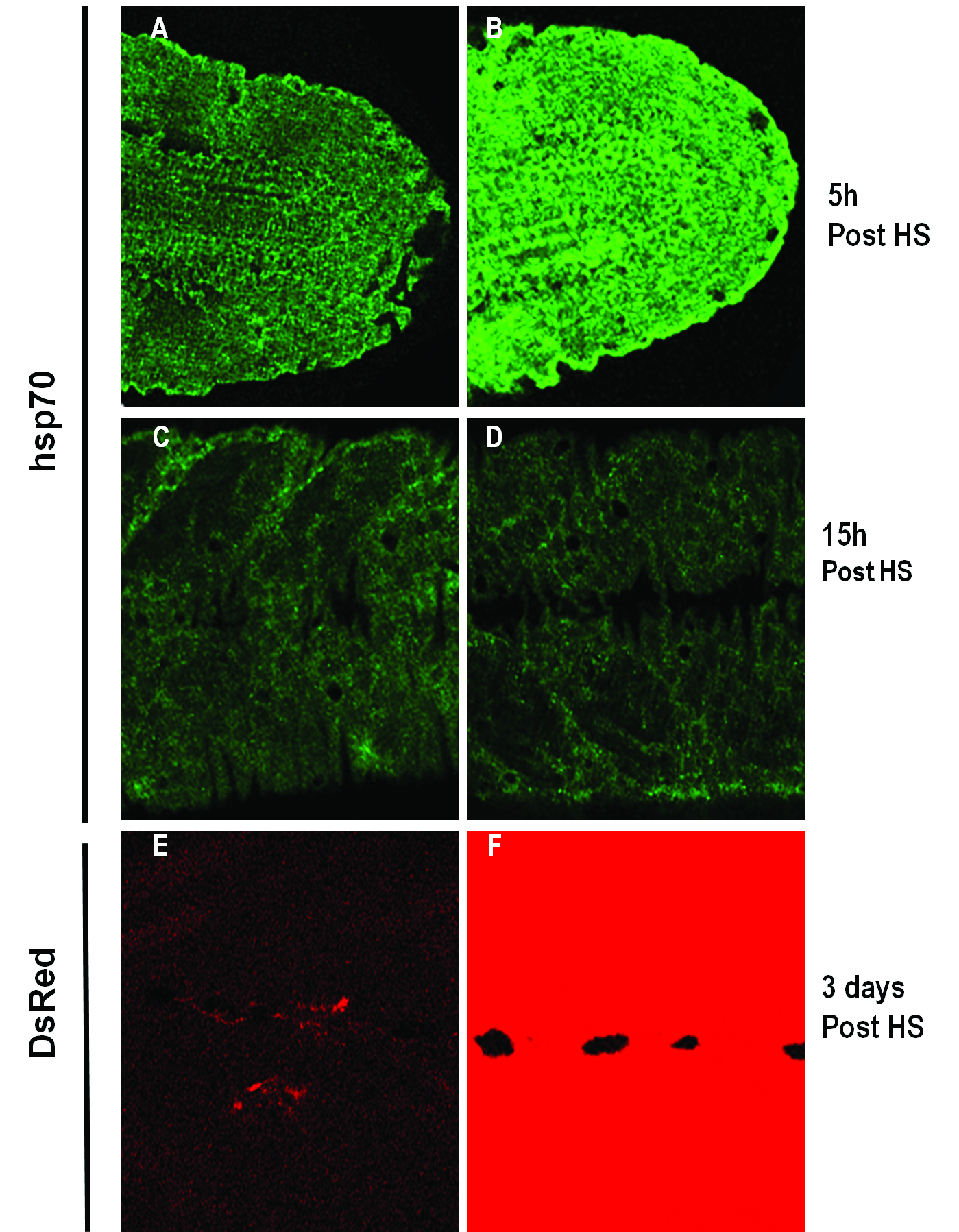

Supplement: Supplementary file 1 [file ana0073-0246-SD1.tif]

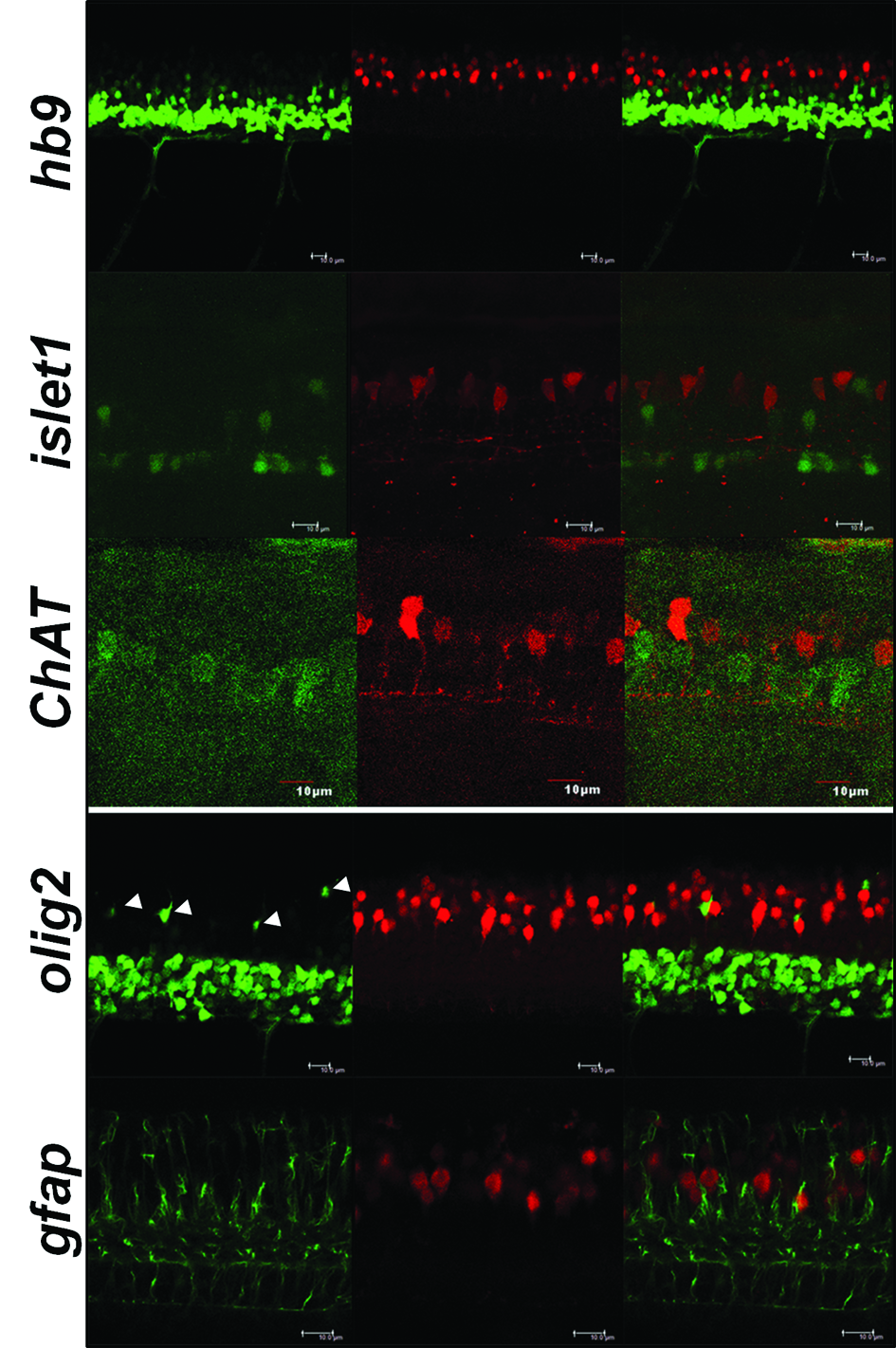

Supplement: Supplementary file 2 [file ana0073-0246-SD2.tif]

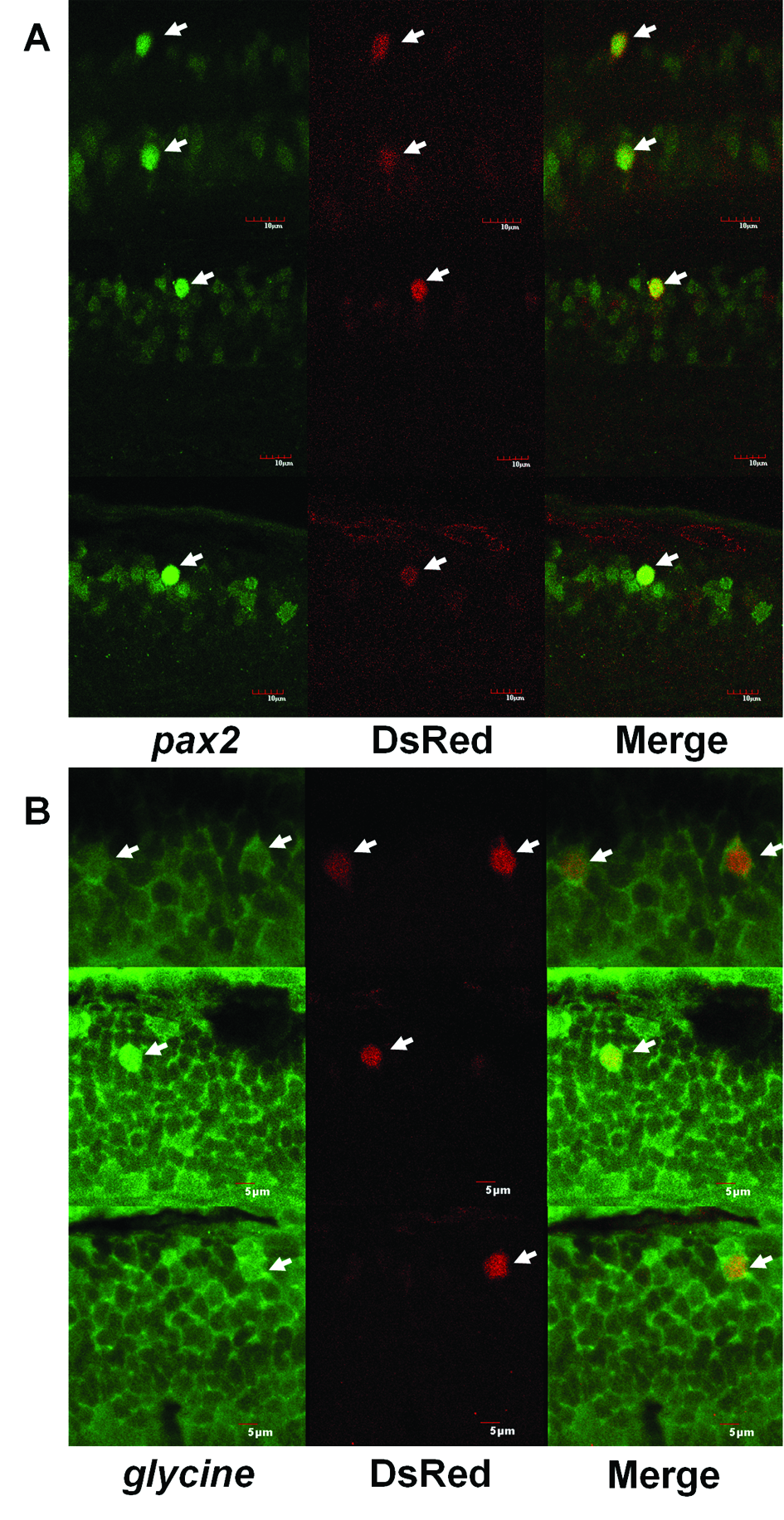

Supplement: Supplementary file 3 [file ana0073-0246-SD3.tif]

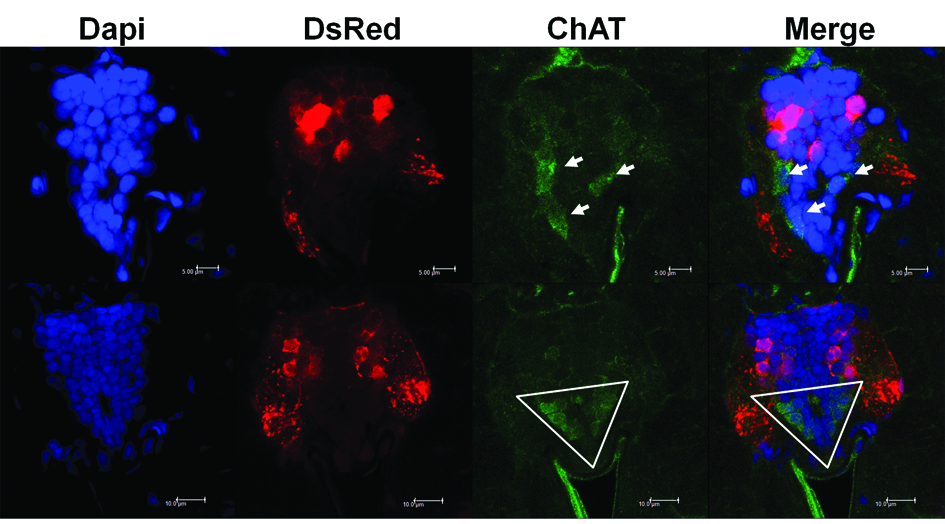

Supplement: Supplementary file 4 [file ana0073-0246-SD4.tif]

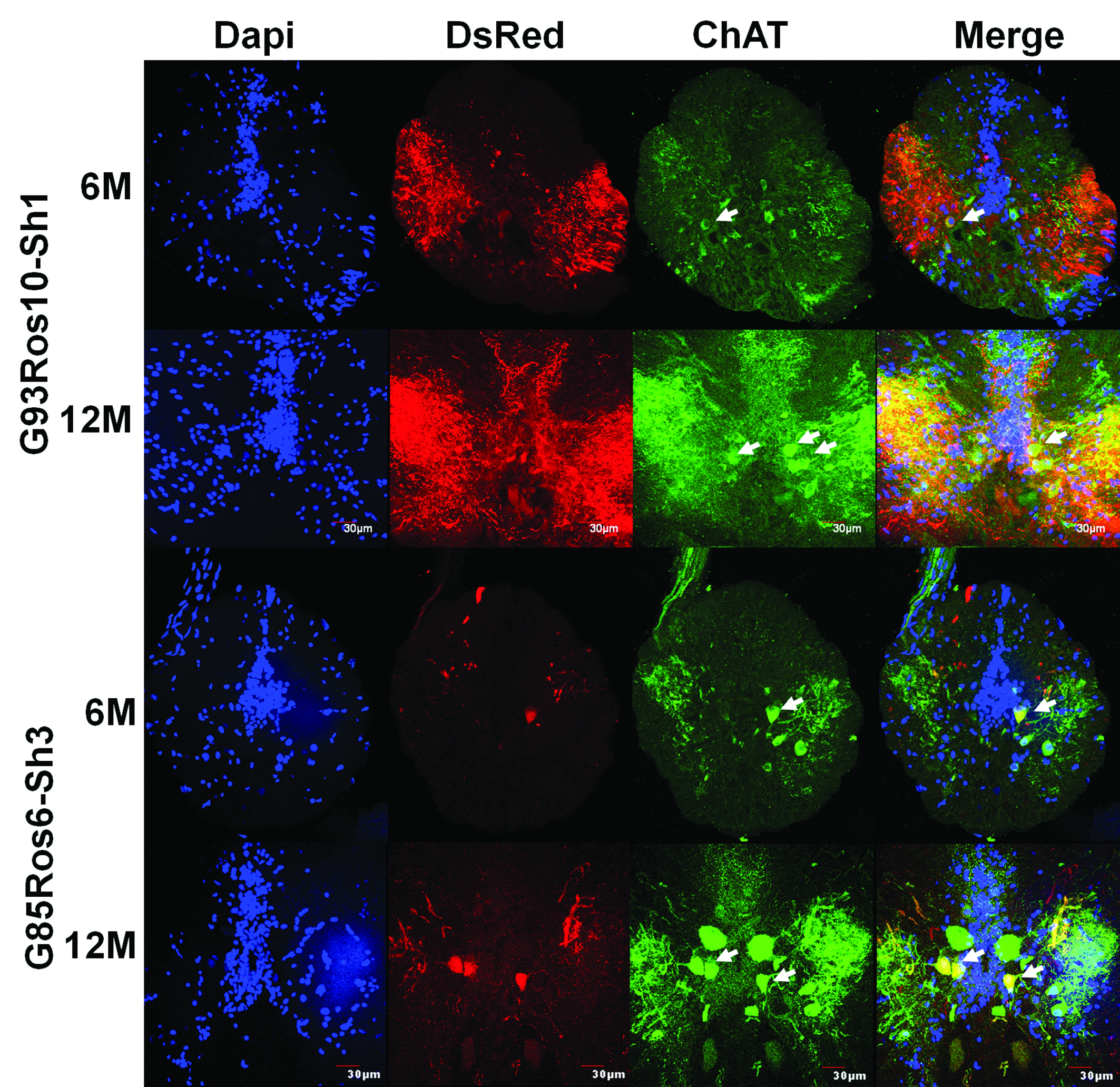

Supplement: Supplementary file 5 [file ana0073-0246-SD5.tif]
