# Supplementary material for: Early Interneuron Dysfunction in ALS: Insights from a Mutant sod1 Zebrafish Model
Source: Ann Neurol. 2012 Dec 31;73(2):246–58. doi: 10.1002/ana.23780 (PMC3608830; doi:10.1002/ana.23780)
Supplement: Supplementary file 6 [file ana0073-0246-SD6.doc]

**Supplementary material-1:**

**Materials and Methods**

***Animals:***

The transgenic lines that were initially developed at the Ohio State University were imported to the University of Sheffield. All adults that were imported were kept in quarantine and the embryos obtained were bleached at 24 hpf and then transferred to the University of Sheffield, Medical Research Council zebrafish facility. Adult and larvae zebrafish (*Danio rerio*) were maintained at 28.5°C and bred according to established procedures [^49^](#_ENREF_49). Animal protocols were undertaken in line with a Home Office approved project licence. The care and maintenance of animals were performed under the Home Office project licence as per ASPA regulations.

***Transgene construct:***

The zebrafish *sod1* transgenic fish were created according to protocols described previously by Ramesh et al.[^13^](#_ENREF_13). For all the transgenic strains, a suffix Sh was added (to imply Sheffield strain) so as to track the source of fish when distributed to other laboratories. The transgenic lines utilized for this study included the *Tg(sod1: sod1^WT^;hsp70:DsRed)os4-Sh4*, the line expressing the highest level of WTSod1 (3.3x as compared to non-transgenics), referred to as WTos4-Sh4 line; *Tg(sod1: sod1^G93R^;hsp70:DsRed)os10-Sh1* referred to as *G93Ros10-Sh1 (*High expressor with Sod1 expression increased 3X and comparable to WTos4-Sh4*), Tg(sod1: sod1^G93R^;hsp70:DsRed)os10-Sh2 referred to as G93Ros6-Sh2 (*Moderate expressor with Sod1 expression increased 2.5X*)* and; *Tg(sod1: sod1^G85R^;hsp70:DsRed) os6-Sh3* line, referred to as G85R*os6-Sh3* (Low expressor with Sod1 expression increased 1.5X). When both G93R and G85R lines are discussed they are referred to as MUT*sod1* lines.

***Heat shocking:***

At 24 hpf, the embryos were heatshocked for 2 hours with 2 cycles (23^0^C for 30 min followed by 37^0^C for 30 minutes) of heating and cooling in a standard thermocycler (Bio-rad Peltier Thermal Cycler PTC-100) with 2 embryos in each tube filled with embryo medium. After heatshocking, the embryos were transferred into petri dishes with embryo medium and incubated at 28.5^0^C. The embryos were examined at 5 h, 15h or 24 h post heat shocking under a fluorescence microscope and fish that showed red fluorescence were identified as transgenic.

**Electrophysiology**

Whole cell voltage clamp recordings were conducted as previously described[^16^](#_ENREF_16). Briefly, 4d larvae were anesthetized and pinned into a Sylgard-lined dish containing 0.02-0.04% tricaine (MS-222, Sigma) dissolved in Evans physiological saline (constituents in mM: 134 NaCl, 2.9 KCl, 2.1 CaCl_2_, 1.2 MgCl_2_, 10 HEPES, and 10 glucose, osmolarity 280–290 mosM, pH 7.8). The skin overlying myotomal tissue was removed with a pair of fine forceps. Subsequently, the tricaine was washed off and fish were perfused with Evans physiological saline containing the neuromuscular blocker D-tubocurarine (10µM), the sodium channel blocker tetrodotoxin (TTX, to synaptically isolate neurons), kynurenic acid (2.5mM, to block spontaneous glutamatergic currents) and bicuculline (25µM, to block spontaneous GABAergic currents). To gain access to the spinal cord, muscle fibres were removed from a two somite region at the level of the yolk extension via aspiration with a broken micropipette. Patch pipettes (3-6MΩ) were fabricated with a P-80 micropipette puller (Sutter Instrument, Novato, CA, USA) and filled with a potassium-gluconate based internal solution (containing in mM: KGluconate 126, KCl 6, NaCl 10, HEPES 10, EGTA 10, MgCl_2_ 2, pH 7.2 pH). Signals were amplified with an RK-400 (BioLogic, Claix, France) patch clamp amplifier and data digitized with a BNC2090 (National Instruments, USA) A-D converter connected to a PC running WinEDR.(**Error! Hyperlink reference not valid.**). Raw signals were acquired at a sample rate of 10 kHz and filtered at 1 kHz.

During experiments series resistance was routinely compensated by 70%. Cells were voltage clamped at -75 mV, a potential at which the chloride-conducting glycine receptors generate inward currents. To determine whether recorded cells were motor neurons, sulforhodamine (0.1%) was included in the electrode solution and upon completion of experiments cells were visually identified with fluorescence microscopy. For consistency, only the most dorsal motor neurons (input resistance <150 MΩ) were used for analysis.

Data were analysed offline with WinEDR. mPSC frequencies were determined by averaging the number of events in a 300 second period. To examine rise time, decay and amplitude of mPSCs, the first 50 mPSCs were selected from each recording and averaged across each experimental condition.

***Immunofluorescence:***

Whole mount staining for synaptic vesicle 2 (SV2-a presynaptic marker) and α-bungarotoxin (BTX-a post synaptic neuromuscular junction marker), was performed as described by Ramesh et al [^13^](#_ENREF_13). ChAT, Pax-2, anti-glycine staining protocols were performed similarly, except that for glycine staining the samples were fixed in paraformaldehyde/gluteraldehyde fixative. The zebrafish embryos or larvae were fixed overnight at 4ºC and permeabilized by successive incubations in dH_2_O and ice cold acetone. The samples were incubated in phosphate buffer containing 1% bovine serum albumin, 1%DMSO and 0.5% Triton-X100 (PBDT) with 5% normal goat serum or normal donkey serum as appropriate, for 60 min, followed by incubation with the appropriate primary antibody from 1 to 3 days at 4^0^C. The samples were washed in PBDT and incubated with species specific fluorescence labelled secondary antibody at 4°C overnight (ON). The samples were then washed with PBDT and mounted on to slides with Vectashield hardest with or without DAPI (Vector Labs) and imaged using confocal microscopy (Olympus). In the case of samples from adult fish, the adult zebrafish were terminally anesthetized in tricaine and decapitated. Muscle and spinal cord were collected and fixed in 4% paraformaldehyde (PFA) ON at 4ºC. The tissues were embedded in O.C.T. (Tissue-Tek) and snap frozen in isopentane. Serial cryostat sections (20 μm) of the tissue were collected on superfrost plus slides (ColePalmer) and stored at -80ºC until processed further as described above. The specific antibodies used in this study were: Alexa-488 conjugated α-bungarotoxin (1:100, Molecular Probes), mouse monoclonal anti-SV-2 antibody (1:50, Developmental Studies Hybridoma Bank), rabbit anti-DsRed antibody (1:100, Clontech) , mouse monoclonal Hsp70 antibody (Thermo Scientific), goat polyclonal anti-ChAT antibody (1:100, Chemicon International) , rabbit polyclonal Pax-2 antibody (1:200, Millipore) and rabbit polyclonal anti-glycine antibody (1:100, Millipore). In *pax-2* and glycine colocalization with DsRed positive neurons in the spinal cord, we could not amplify the DsRed signal with DsRed antibody, as polyclonal rabbit anti-Pax-2 antibody was used. Hence the primary DsRed fluorescence signal (although weak) was used to identify colocalization. The secondary antibodies utilized were: Alexa-633 conjugated goat anti-mouse antibody, Alexa-633 conjugated goat anti-rabbit antibody, Alexa-633 conjugated Donkey anti-goat antibody, Alexa-568 conjugated goat anti-rabbit antibody and Alexa-568 conjugated donkey anti-rabbit antibody.

Quantitative analysis of confocal images was performed on image stacks of 16-20 μm thickness (0.5-1μm /section) obtained and processed using Image J Software (National Institutes of Health). For quantitation of the fluorescence intensity of individual neurons, an outline around the fluorescent cells was drawn and the average fluorescence intensity measured. A minimum of 50 DsRed positive neurons from multiple larval samples were pooled for analysis in quantifying the average DsRed fluorescence of G93Ros6-sh2 and WTos4-sh4 lines. Image analysis for NMJ analysis was performed using NIH ImageJ software and quantitative analysis of the NMJ was performed using a colocalization analysis plugin.

**Fluorescent RNA *in situ* hybridisation and quantification of cells**

Fluorescent RNA *in situ* hybridisation was performed on 24 hpf G93Ros10 embryos fixed in 4% PFA, as previously described[^17^](#_ENREF_17). The plasmids were kindly provided by K. Lewis (Syracuse University). A mix of equal concentrations of the probes (glyt2a, glyt2b and DsRed), (gad65,67 and DsRed) or (vglut2 and DsRed) was used as previously described. Quantification of cells was done by counting DsRed/glycine, DsRed/GABA and DsRed/vglut2 positive cells in the mid-trunk region and the percentage of DsRed cells showing glycine, GABA or glutamate staining, as well as the percentage of each cell types showing DsRed staining was calculated from an average of 10 embryos (a total of 429, 505 and 634 DsRed cells were counted respectively for each riboprobe pair).

***Fluorimetry:***

For fluorimetry using larval lysates, the zebrafish larvae were deeply anesthetized with tricaine. The embryos were sorted as transgenic or non-transgenic and 3 embryos/tube were placed in Eppendorf tubes. Liquid was removed and 100 μL of PBS was added. The larvae were lysed by pulsed sonication at 75% of full vibration amplitude (15” pulses, Misonix Sonicator 4000) on ice until thoroughly homogenized. Lysates were then centrifuged at maximum speed for 5 minutes. 50 μL of supernatant was analyzed in a 96-well plate (Corning-3880, Corning Life Sciences) at an excitation wavelength of 530 nm and an emission wavelength of 580 nm using a FLEX Station plate reader (Molecular Devices) and analyzed with SOFTmax Pro Software (Molecular Devices). Measurements were reported as mean ± s.e.m. after subtraction of background fluorescence from non-transgenic samples.

***Drug testing:***

The G93Ros10-Sh4 line was used to identify drugs that inhibit neuronal stress. 24 hpf embryos obtained by timed matings, were dechorionated. The embryos were put in plates (25-50 embryos/plate) containing the appropriate dose of the test compound in embryo medium. The drug-containing media was changed daily and maintained for 5 days. At 5 dpf, the embryos were sorted as transgenic or non-transgenic and put in tubes with 3 embryos/tube and sonicated in 100µl phosphate buffer saline (PBS). The samples were then centrifuged at 3000G for 10 minutes. The supernatant (75µl) was added to 96 well fluorescent clear bottom plates and the well fluorescence was measured using FLUOstar Omega (415-0153) from BMG Labtech, Offenburg, Germany. The controls used in drug studies were solvent alone. The treatment effect was measured by ANOVA and compared to vehicle treated samples and the IC50 was calculated with a dose range study. To compare across different experiments, the percent inhibition of the fluorescence signal by test compounds as compared to vehicle in each study was used for standardization. Riluzole (10µM), a drug used clinically for neuroprotection in ALS, was used as a positive control for validation of the assay. The other compounds tested R-apomorphine and Tricaine (Tricaine methanesulfonate) were added at 10µM and 610 µM doses respectively.

***Statistical analysis:***

Statistics were performed using SPSS 10. The values from the individual images were compared between control and transgenic lines using an unpaired t-test or ANOVA with post-hoc Bonferroni testing to compare groups. Electrophysiological data were compared using the two way Kolmogorov–Smirnov test.
